# Effects of 3-(4-Hydroxy-3-methoxyphenyl)propionic Acid on Regulating Oxidative Stress and Muscle Fiber Composition

**DOI:** 10.3390/nu17040668

**Published:** 2025-02-13

**Authors:** Yishan Tong, Sihui Ma, Riyo Awa, Takashi Tagawa, Yasuhiro Seki, Tiehan Cao, Haruki Kobori, Katsuhiko Suzuki

**Affiliations:** 1 Graduate School of Sport Sciences, Waseda University, Tokorozawa 359-1192, Japan; tongyishan130@ruri.waseda.jp (Y.T.); y-seki@wismerll.co.jp (Y.S.); caotiehan0313@toki.waseda.jp (T.C.); koboharu1223@fuji.waseda.jp (H.K.); 2 Faculty of Human Sciences, Waseda University, Tokorozawa 359-1192, Japan; masihui@toki.waseda.jp; 3 Research Center, Maruzen Pharmaceuticals Co., Ltd., Fukuyama 729-3102, Japan; r-awa@maruzenpcy.co.jp (R.A.); t-tagawa@maruzenpcy.co.jp (T.T.); 4 Faculty of Sport Sciences, Waseda University, Tokorozawa 359-1192, Japan

**Keywords:** 3-(4-hydroxy-3-methoxyphenyl)propionic acid, dihydroferulic acid, oxidative stress, muscle fiber composition, gut microbiota-derived metabolite

## Abstract

**Background/Objectives**: Our previous study demonstrated that 3-(4-hydroxy-3-methoxyphenyl)propionic acid (HMPA) administration improved grip strength and reduced blood urea nitrogen levels, but its underlying mechanisms remain unclear. This study aimed to investigate the effects of HMPA on oxidative stress and muscle fiber composition, emphasizing its potential role in modulating redox signaling pathways and influencing muscle development. **Methods**: Eight-week-old male C57BL/6 mice were orally administered HMPA solution (50 or 500 mg/kg/day) or distilled water (10 mL/kg) for 14 days, and then divided into sedentary and exhaustive exercise groups to evaluate oxidative stress status, myosin heavy chain (MHC) isoform expression, and underlying mechanisms. **Results**: Both low and high doses of HMPA reduced oxidative stress by decreasing plasma reactive oxygen metabolites. High-dose HMPA reduced plasma nitrite/nitrate levels and enhanced antioxidant capacity post-exercise, accompanied by changes in the mRNA abundance of antioxidant enzymes (e.g., *Sod1* and *Nqo1*) and reductions in the mRNA abundance of nitric oxide synthases (e.g., *Nos2* and *Nos3*) in the soleus. Additionally, high-dose HMPA administration increased the protein expression of MYH4 in the soleus, while low-dose HMPA enhanced the gene expression of *Myh4* and *Igf1*, suggesting that HMPA may promote fast-twitch fiber hypertrophy through the activation of the IGF-1 pathway. Furthermore, low-dose HMPA significantly increased the gene expression of *Sirt1* and *Nrf1*, as well as AMPK phosphorylation post-exercise, suggesting low-dose HMPA may improve mitochondrial biogenesis and exercise adaptation. **Conclusions**: These findings suggest that HMPA may serve as a dietary supplement to regulate redox balance, enhance antioxidant defenses, and promote the formation of fast-twitch fibers.

## 1. Introduction

Skeletal muscle, which constitutes approximately 40% of the total human body mass, primarily functions to generate contractile force for supporting posture, movement, and breathing, while contributing to metabolic processes such as thermogenesis [1,2]. The contractile and metabolic properties of skeletal muscle are determined by its fiber type composition, which is classified into fast-twitch (Type II) and slow-twitch (Type I) fibers based on myosin heavy chain (MHC) isoforms [3]. MHC isoforms include four primary types: MHC IIx, MHC IIa, MHC IIb, and MHC I, corresponding to the genes *Myh1*, *Myh2*, *Myh4*, and *Myh7*, respectively [4,5]. Fast-twitch fibers, which express MHC II isoforms, can be further divided into Type IIa, IIx, and IIb, with a sequential increase in glycolytic properties, muscle contractile velocity, and susceptibility to fatigue [6,7]. In contrast, slow-twitch fibers, which express MHC I, exhibit high oxidative capacity and excellent fatigue resistance but generate lower contractile force compared to fast-twitch fibers [5]. Skeletal muscle is a highly plastic tissue that adapts to internal and external stimuli by altering its mass, fiber type composition, metabolic properties, mitochondrial content, and capillarization [8,9,10].

Redox signaling is essential for muscle fiber growth, repair, and functional adaptation [11,12]. As signaling messengers in redox pathways, reactive oxygen species (ROS) and reactive nitrogen species (RNS) are highly reactive molecules containing oxygen or nitrogen atoms, including hydrogen peroxide (H_2_O_2_), superoxide anion (O_2_^−^), singlet oxygen (^1^O_2_), nitric oxide (NO), hydroxyl radical (•OH), and peroxyl radical (•O_2_) [13]. It has been widely documented that the production of ROS and RNS is indispensable for supporting normal cellular processes, facilitating mitochondrial biogenesis, angiogenesis, and antioxidant enzyme activation, all of which are important for endurance-induced muscle adaptation [14,15]. However, the excessive production of ROS and RNS leads to oxidative stress and contributes to the pathogenesis of muscle decline and atrophy, such as age-related and malnutrition-related muscle atrophy [16,17]. Exhaustive exercise can lead to oxidative stress by triggering the excessive production of ROS [18,19]. The accumulation of ROS exacerbates inflammation and muscle damage, which further causes fatigue and compromises skeletal muscle-related exercise performance and recovery [18,19]. To prevent the resulting oxidative stress, the endogenous antioxidant defense system neutralizes harmful molecules through the regulation of key antioxidant enzymes, including superoxide dismutase (SOD), catalase (CAT), and glutathione peroxidase (GPx) [20].

Natural polyphenolic antioxidants have emerged as promising intervention strategies to modulate the redox status of muscle tissue, thereby influencing muscle fiber composition and providing protection against oxidative stress-induced damage [21,22,23]. The gut microbiota is essential for converting polyphenols into smaller, more absorbable metabolites, which support a range of health benefits [24]. One such prominent metabolite is 3-(4-hydroxy-3-methoxyphenyl)propionic acid (HMPA, dihydroferulic acid), which is primarily derived from the microbial metabolism of dietary polyphenols, including 4-hydroxy-3-methoxycinnamic acid (HMCA, ferulic acid) [25], curcumin [26], and chlorogenic acid (CGA) [27], as well as polyphenol-rich foods such as whole-grain wheat [28], tea [29], and coffee [30]. Moreover, HMPA can also be naturally produced during the fermentation of Kurosu (Japanese unpolished rice vinegar) and wolfberry juice, contributing to the antioxidant properties of these foods [31,32]. Microbial metabolites have the potential to retain the biological activity of their parent compounds and may even exhibit enhanced bioactivity [24,31,33,34]. For example, as a metabolite derived from HMCA, HMPA has been shown to exhibit anti-inflammatory properties comparable to those of its precursor and, in some cases, to demonstrate stronger antioxidant effects [31,33].

The potential role of polyphenol antioxidants in reducing oxidative stress and improving skeletal muscle function is attributed to their anti-inflammatory and antioxidant properties [18,22,23,25]. HMCA has been extensively studied for its beneficial effects on skeletal muscle function [18,22,35]. It has been reported to induce muscle hypertrophy and promote the hypertrophic growth of either fast-twitch or slow-twitch fibers in different animal models [22,35]. Additionally, HMCA has been shown to enhance exercise endurance capacity and protect against exercise-induced fatigue by activating antioxidant defenses and reducing oxidative stress and inflammation [18]. Recent studies have demonstrated that the benefits of HMCA are, in part, attributed to its gut microbiota-derived metabolite, HMPA, which has attracted increasing attention for its potential application in enhancing muscle function [25,34]. Moreover, HMPA, being a low-toxicity natural byproduct, offers certain practical advantages due to its superior stability and higher absorption rate compared to HMCA [30,34]. HMPA has been widely recognized for its antioxidant and anti-inflammatory properties in numerous studies, providing protective effects against exogenous agent-induced oxidative stress, but its impact on skeletal muscle remains to be further explored [33,36]. Previous research showed that HMPA mitigated dexamethasone-induced reductions in myotube diameter and muscle protein breakdown [37]. In our previous study, a 14-day administration of HMPA improved muscle strength and reduced plasma blood urea nitrogen levels, indicating that HMPA has the potential to preserve muscle mass and enhance muscle function [38]. However, the underlying mechanisms remain to be elucidated. Building on these findings, we hypothesize that HMPA influences oxidative stress levels and muscle fiber type transformation, thereby enhancing muscle strength and mitigating oxidative stress damage caused by exhaustive exercise. This study aims to (1) examine the antioxidative properties of HMPA and its effects on mitigating oxidative stress, and (2) investigate its potential to regulate muscle fiber composition and promote muscle adaptation.

## 2. Materials and Methods

### 2.1. Study Design

The study design and animals employed in this work were consistent with those detailed in our previous study, which assessed the effects of HMPA on skeletal muscle function through grip force and treadmill exhaustion tests [38]. The HMPA formulation, containing 25.4% HMPA combined with dextrin, was provided by Maruzen Pharmaceuticals Co., Ltd. (Hiroshima, Japan). In brief, eight-week-old male C57BL/6 mice (*n* = 48) were orally administered HMPA solution (50 or 500 mg/kg body weight/day) or vehicle (distilled water) at a gavage volume of 10 mL/kg for 14 consecutive days. Two days prior to the final administration, all the mice were acclimated to a treadmill running at 15 m/min for 10 min without an incline. After treadmill acclimatization, the mice were subdivided into sedentary (SED) and exhaustive exercise (EX) groups as follows: (1) control administration + SED (C) group, (2) control administration + EX (CE) group, (3) low-dose HMPA administration + SED (L) group, (4) low-dose HMPA administration + EX (LE) group, (5) high-dose HMPA administration + SED (H) group, (6) high-dose HMPA administration + EX (HE) group. The sample size (*n* = 8) per group was determined based on previous sample size calculations conducted by Graber et al. [39] and our prior research [40] to ensure sufficient statistical power.

The treadmill exhaustion test was conducted one hour after the final oral administration, starting at a 7° incline and 10 m/min for 15 min, with speed increases to 15 m/min and 20 m/min, each lasting 15 min. Finally, the speed increased to 24 m/min until exhaustion, which was defined as the inability to maintain the speed and frequent turning despite persistent prodding with a brush. After completing all the tests, plasma and muscle samples (gastrocnemius and soleus) were immediately collected, flash-frozen in liquid nitrogen, and stored at −80 °C in an ultralow-temperature freezer until further analysis. All the animal care procedures followed the Guiding Principles for the Care and Use of Animals established by the Waseda University Institutional Animal Care and Use Committee, and the study protocol was approved under number A23-127.

### 2.2. Assessment of Antioxidant Capacity and Oxidative Stress Biomarkers in Plasma

Plasma oxidative stress levels and antioxidant capacity were assessed using diacron-reactive oxygen metabolites (d-ROMs), biological antioxidant potential (BAP), and OXY-adsorbent test kits (Wismerll Co., Ltd., Tokyo, Japan) with a Free Radical Elective Evaluator (FREE; Wismerll Co. Ltd., Tokyo, Japan). Plasma nitrite/nitrate concentration were measured using the Griess Reagent System Kit (Promega, Madison, WI, USA) and a SpectraMax iD5 microplate reader (Molecular Devices, San Jose, CA, USA). All the procedures followed the manufacturer’s protocol.

#### 2.2.1. d-ROMs Test

The d-ROMs test was used to assess reactive oxygen metabolites by quantifying the levels of organic hydroperoxides, which are metabolites formed when ROS oxidize biological molecules (e.g., lipids, proteins, and DNA). The plasma samples (20 μL) were mixed with an acetate buffer solution (pH 4.8), facilitating iron ions to catalyze the conversion of peroxides into free radicals. Subsequently, 20 μL of a chromogenic solution containing N,N-Diethyl-p-phenylenediamine was added, which reacts with the radicals to form a colored solution. The color intensity was quantified spectrophotometrically at 505 nm, and the outcomes were expressed in U.CARR, where 1 U.CARR corresponds to 0.08 mg H_2_O_2_/dL.

#### 2.2.2. BAP Test

The BAP test was performed to measure the antioxidant capacity of the plasma by evaluating its ability to reduce ferric ions (Fe^3+^) to ferrous ions (Fe^2+^). The plasma samples (10 μL) were reacted with ferric chloride (FeCl_3_) and a thiocyanate reagent to generate a red color, indicating the presence of Fe^3+^ ions. The reduction of Fe^3+^ to Fe^2+^ caused decolorization, and this change in color was detected spectrophotometrically at 505 nm, with the results recorded in μmol/L.

#### 2.2.3. OXY-Adsorbent Test

The OXY-adsorbent test was used to assess the total antioxidant status of the plasma by measuring its ability to neutralize hypochlorous acid (HClO), a highly reactive oxygen species. The plasma samples (10 μL) were mixed with HClO and incubated for 10 min. Then, 10 μL of a chromogenic solution containing N,N-Diethyl-p-phenylenediamine was added to react with the remaining HClO, resulting in a red color reaction. The OXY-adsorbent test values were expressed as μmol/mL HClO.

#### 2.2.4. Griess Reagent System

The Griess Reagent System assay kit was used to measure the plasma nitrite/nitrate concentration, which serves as an indicator of nitric oxide synthase (NOS) activity and nitric oxide production. In brief, 50 µL of each plasma sample was mixed with an equal volume of sulfanilamide solution and allowed to react for 5–10 min under light-protected conditions. Subsequently, 50 µL of N-1-naphthylethylenediamine dihydrochloride solution was introduced, followed by an additional 5–10 min incubation period. After the incubation period, absorbance was measured at 540 nm, and nitrite/nitrate concentration was quantified using a standard curve, with the values expressed in μmol/L.

### 2.3. Assessment of Lipid Peroxidation in Soleus and Gastrocnemius

Lipid peroxidation was assessed by measuring the malondialdehyde (MDA) content using a TBARS Assay Kit (Cayman Chemical, Ann Arbor, MI, USA) according to the manufacturer’s instructions.

### 2.4. RNA Extraction and Quantitative PCR (qPCR) Analysis

Total RNA was extracted from gastrocnemius and soleus using the NucleoSpin RNA Kit (Takara Bio, Shiga, Japan). The concentration and purity of the extracted RNA were evaluated with the NanoDrop ND-1000 spectrometer (NanoDrop Technologies, Wilmington, DE, USA). The RNA was reverse transcribed into complementary DNA (cDNA) using the PrimeScript™ FAST RT reagent Kit with gDNA Eraser (Takara Bio Inc., Shiga, Japan). PCR amplification was carried out on the Thermal Cycler Dice^®^ Real-Time System IV (Takara Bio, Shiga, Japan) using TB Green^®^ Premix Ex Taq™ II (Fast qPCR) (Takara Bio, Shiga, Japan) in adherence to the manufacturer’s instructions. Ribosomal protein S18 (*Rps18*) was selected as a reference gene. The relative expression levels of the target genes were calculated using the 2^−∆∆Ct^ method. The primer sequences for each target gene are listed in Table 1.

### 2.5. Western Blot Analysis

Proteins were extracted from the gastrocnemius and soleus using lysis buffer containing pierce protease and phosphatase inhibitor mini-tablets, EDTA-free (Thermo Fisher Scientific, Waltham, MA, USA). Protein concentrations were measured using the BCA method, and the samples were heated at 95 °C for 10 min in prepared Laemmli Sample Buffer (Bio-Rad, Hercules, CA, USA). The protein samples were equally loaded onto 8% SDS-polyacrylamide gels for electrophoretic separation and transferred onto PVDF membranes (Millipore, Bedford, MA, USA).

The membranes were blocked with 5% bovine serum albumin (BSA) for 1 h at room temperature and incubated overnight at 4 °C with primary antibodies, including AMP-activated protein kinase α (AMPKα, #5831), phospho-AMPKα (Thr172, #2535), the mechanistic target of rapamycin (mTOR, #2983), and phospho-mTOR (Ser2448, #5536). All the antibodies were sourced from Cell Signaling Technology (Beverly, MA, USA). Total MHC (MF20) and MHC IIb (BF-F3) antibodies were acquired from the Developmental Studies Hybridoma Bank (Iowa City, IA, USA), and MHC I (MYH7) (sc53090) was obtained from Santa Cruz Biotechnology (Dallas, TX, USA). All the primary antibodies were used at a 1:1000 dilution, except alpha-tubulin (Proteintech, Rosemont, IL, USA), which was applied at a 1:5000 dilution.

Secondary antibodies (goat anti-rabbit IgG or goat anti-mouse IgG; Cell Signaling Technology) were applied for 1 h at room temperature. Signals were detected using the FujiFilm LAS-300 image reader (FujiFilm, Tokyo, Japan), and the grayscale intensity of the protein bands was quantified using the ImageJ software (version 1.54g, NIH, Bethesda, MD, USA). The phosphorylation levels of mTOR and AMPK were determined by calculating the ratio of the phosphorylated protein band intensity to the corresponding total protein band intensity. The expression levels of the target proteins were normalized to alpha-tubulin.

### 2.6. Statistical Analysis

Statistical analysis was conducted using GraphPad Prism (version 10.1.0, GraphPad Software, La Jolla, CA, USA). All the data were expressed as the mean ± standard error of the mean (SEM). A two-way ANOVA followed by Tukey’s post hoc test was performed to evaluate group differences. Tukey’s post hoc test was chosen for its suitability for equal sample sizes, its ability to control Type I error rates, and its effectiveness in ensuring reliable and interpretable results. If a significant interaction was found, simple main-effects analysis and Tukey’s post hoc test were used to identify specific differences. If no significant interaction was found, but the main effects were significant, main-effects analysis and Tukey’s post hoc test were applied to assess group differences. Statistical significance was set at *p* < 0.05, while a trend was considered when 0.05 ≤ *p* ≤ 0.10.

## 3. Results

### 3.1. Effects of HMPA Administration on Oxidative Stress and Antioxidant Capacity in Plasma

**Figure 1:**
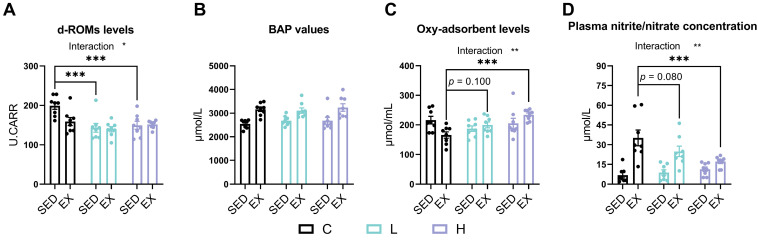
(**A**) d-ROMs test, (**B**) BAP test, (**C**) OXY-adsorbent test, and (**D**) the nitrite/nitrate concentration were assessed in mice plasma. d-ROMs, diacron-reactive oxygen metabolites; BAP, biological antioxidant potential. C group, vehicle control group; L group, low-dose HMPA administration group; H group, high-dose HMPA administration group; SED, sedentary; EX, exercise. The values are expressed as the mean ± SEM; Interaction: interactive effect between HMPA administration and exercise; * *p* < 0.05, ** *p* < 0.01, and *** *p* < 0.001 compared with the C group.

The d-ROMs test results showed that HMPA administration significantly decreased oxidative stress levels (Figure 1A, *p* < 0.001), while a trend toward a main effect of exercise (Figure 1A, *p* = 0.060) was observed. A significant interaction effect between HMPA administration and exercise was also observed (Figure 1A, *p* < 0.05). The post hoc analysis indicated that compared to the C group, d-ROM levels significantly decreased in both the L group (Figure 1A, *p* < 0.001) and H group (Figure 1A, *p* < 0.001). These results suggest that HMPA administration significantly reduces oxidative stress.

The BAP test results showed that exhaustive exercise significantly increased BAP values (Figure 1B, *p* < 0.0001), but no significant differences were observed following HMPA administration.

In the OXY-adsorbent test, a significant interaction between HMPA administration and exercise was observed (Figure 1C, *p* < 0.01), along with a significant main effect of HMPA administration (*p* < 0.05). The post hoc analysis indicated that the OXY-adsorbent levels in the HE group were significantly higher than those in the CE group (Figure 1C, *p* < 0.001), while low-dose HMPA exhibited an increasing trend without reaching statistical significance (Figure 1C, *p* = 0.100). These results indicate a higher plasma antioxidant capacity in the HMPA-administered groups.

As shown in Figure 1D, the plasma nitrite/nitrate concentration significantly increased after exhaustive exercise (Figure 1D, *p* < 0.0001). There was a significant interaction between HMPA administration and exercise (Figure 1D, *p* < 0.01). The post hoc analysis showed that high-dose HMPA administration significantly reduced the increase in plasma nitrite/nitrate concentration induced by exhaustive exercise (Figure 1D, *p* < 0.001), while low-dose HMPA showed a similar decreasing trend (Figure 1D, *p* = 0.080).

### 3.2. Effects of HMPA Administration on Malondialdehyde Content in Soleus and Gastrocnemius

**Figure 2:**
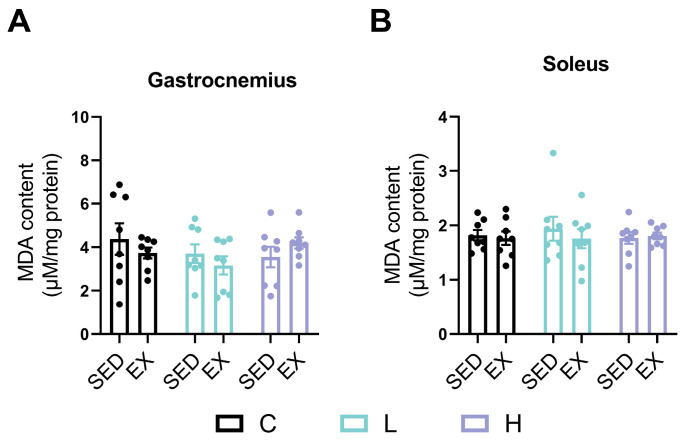
MDA content of (**A**) gastrocnemius and (**B**) soleus. C group, vehicle control group; L group, low-dose HMPA administration group; H group, high-dose HMPA administration group; SED, sedentary; EX, exercise. The values are expressed as the mean ± SEM.

MDA is recognized as the final product of lipid peroxidation, reflecting the extent of oxidative damage in tissues [41]. No significant changes in the MDA content were found in the gastrocnemius and soleus muscles, as shown in Figure 2A,B.

### 3.3. Effects of HMPA Administration on Antioxidant Enzyme Gene Expression in Soleus and Gastrocnemius

**Figure 3:**
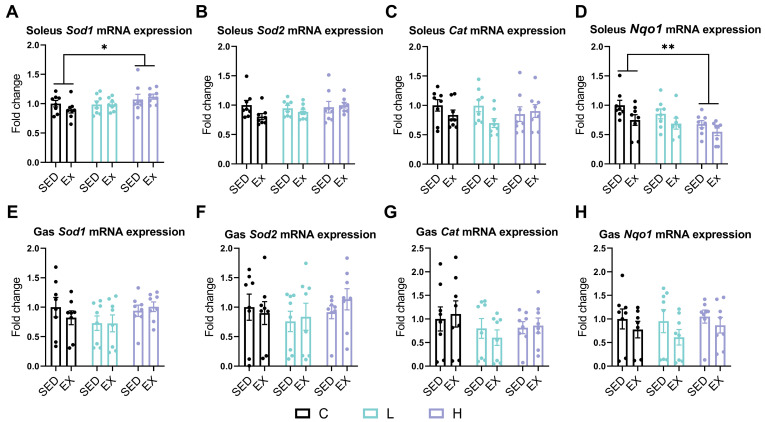
mRNA expression of (**A**) *Sod1*, (**B**) *Sod2*, (**C**) *Cat*, and (**D**) *Nqo1* in the soleus, and (**E**) *Sod1*, (**F**) *Sod2*, (**G**) *Cat*, and (**H**) *Nqo1* in the gastrocnemius. *Sod*, superoxide dismutase; *Cat*, catalase; *Nqo1*, NAD(P)H dehydrogenase, quinone 1; Gas, gastrocnemius; C group, vehicle control group; L group, low-dose HMPA administration group; H group, high-dose HMPA administration group; SED, sedentary; EX, exercise. The values are expressed as the mean ± SEM; * *p* < 0.05 and ** *p* < 0.001 compared with the C group.

To gain deeper insights into the impact of the HMPA administration on the antioxidant defense system, we analyzed the gene expression of key antioxidant enzymes, including *Sod1*, *Sod2*, *Cat*, and NAD(P)H quinone dehydrogenase 1 (*Nqo1*) in mouse soleus and gastrocnemius, respectively. In the soleus, the high-dose HMPA administration significantly increased *Sod1* expression (Figure 3A, *p* < 0.05) but significantly decreased *Nqo1* expression (Figure 3D, *p* < 0.001) compared to the vehicle control administration. Neither exercise nor HMPA administration had any significant effect on the expression levels of *Sod2* (Figure 3B) or *Cat* (Figure 3C) in the soleus.

However, in the gastrocnemius, the expression levels of *Sod1* (Figure 3E), *Sod2* (Figure 3F), *Cat* (Figure 3G), and *Nqo1* (Figure 3H) were not affected by either the HMPA administration or exercise.

### 3.4. Effects of HMPA Administration on Nitric Oxide Synthase Gene Expression in Soleus and Gastrocnemius

**Figure 4:**
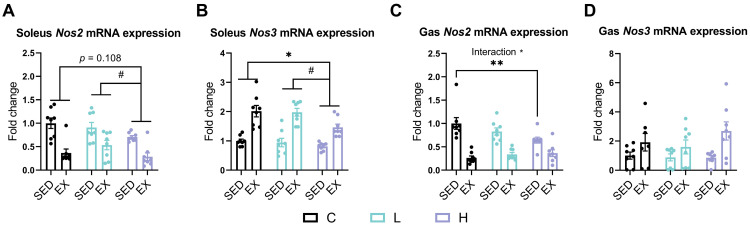
mRNA expression of (**A**) *Nos2* and (**B**) *Nos3* in the soleus. mRNA expression of (**C**) *Nos2* and (**D**) *Nos3* in the gastrocnemius. *Nos2* (*iNOS*), nitric oxide synthase 2, inducible; *Nos3* (*eNOS*), nitric oxide synthase 3, endothelial cell; Gas, gastrocnemius; C group, vehicle control group; L group, low-dose HMPA administration group; H group, high-dose HMPA administration group; SED, sedentary; EX, exercise. The values are expressed as the mean ± SEM. Interaction: interactive effect between HMPA administration and exercise; * *p* < 0.05 and ** *p* < 0.001, compared with the C group; # *p* < 0.05 compared with the H group.

Exhaustive exercise decreased *Nos2* (*iNOS*) expression while increasing *Nos3* (*eNOS*) expression in both the soleus and gastrocnemius (Figure 4). In the soleus, the HMPA administration significantly decreased the expression of both *Nos2* (*iNOS*) (Figure 4A, *p* < 0.05) and *Nos3* (*eNOS*) (Figure 4B, *p* < 0.05). Specifically, *Nos2* (*iNOS*) expression was significantly lower in the high-dose HMPA administration group compared to the low-dose group (Figure 4A, *p* < 0.05), with a trend toward reduction compared to the vehicle control group (Figure 4A, *p* = 0.108). Similarly, *Nos3* (*eNOS*) expression was significantly reduced in the high-dose HMPA group compared to both the low-dose (Figure 4B, *p* < 0.05) and vehicle control (Figure 4B, *p* < 0.05) groups.

In the gastrocnemius, a significant interaction between the HMPA administration and exercise was observed for *Nos2* (*iNOS*) (Figure 4C, *p* < 0.05). The H group showed a significant reduction in *Nos2* (*iNOS*) expression compared to the C group (Figure 4C, *p* < 0.01). However, the HMPA administration did not significantly affect *Nos3* (*eNOS*) expression levels regardless of exercise status (Figure 4D).

### 3.5. Effects of HMPA Administration on Myosin Heavy Chain Isoform Gene Expression in Soleus and Gastrocnemius

**Figure 5:**
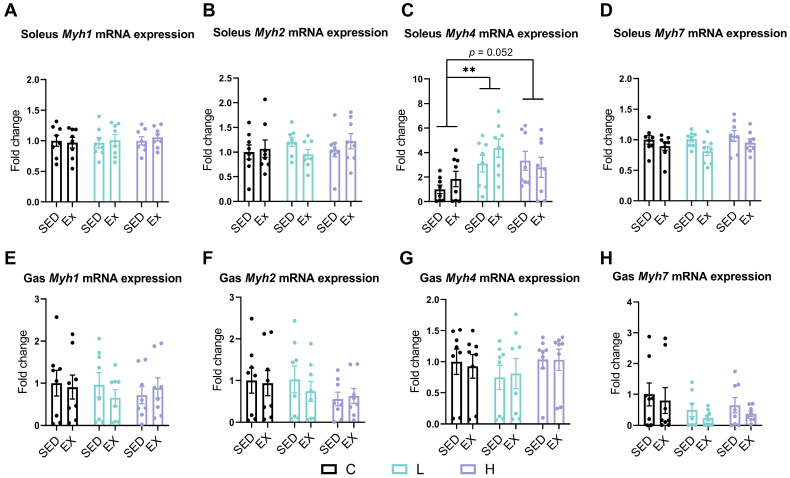
mRNA expression of (**A**) *Myh1*, (**B**) *Myh2*, (**C**) *Myh4,* and (**D**) *Myh7* in the soleus. The mRNA expression of (**E**) *Myh1*, (**F**) *Myh2*, (**G**) *Myh4*, and (**H**) *Myh7* in the gastrocnemius. *Myh1*, myosin, heavy polypeptide 1, skeletal muscle, adult; *Myh2*, myosin, heavy polypeptide 2, skeletal muscle, adult; *Myh4*, myosin, heavy polypeptide 4, skeletal muscle; *Myh7*, myosin, heavy polypeptide 7, cardiac muscle, beta; Gas, gastrocnemius; C group, vehicle control group; L group, low-dose HMPA administration group; H group, high-dose HMPA administration group; SED, sedentary; EX, exercise. The values are expressed as the mean ± SEM; ** *p* < 0.01 compared with the C group.

To investigate the effects of the HMPA administration on muscle fiber composition, we first examined the mRNA expression levels of MHC isoforms, including *Myh1* (encoding MHC IIx), *Myh2* (encoding MHC IIa), *Myh4* (encoding MHC IIb), and *Myh7* (encoding MHC I). Exhaustive exercise did not affect the expression levels of *Myh1*, *Myh2*, *Myh4*, and *Myh7* in both the soleus and gastrocnemius (Figure 5). The low-dose HMPA administration significantly increased *Myh4* expression levels (Figure 5C, *p* < 0.01), and a similar trend was observed with the high-dose HMPA administration (Figure 5C, *p* = 0.052) in the soleus. Neither HMPA administration nor exhaustive exercise changed the gene expression levels of *Myh1* (Figure 5A), *Myh2* (Figure 5B), or *Myh7* (Figure 5D) in the soleus. No significant effects were observed in the expression levels of *Myh1* (Figure 5E), *Myh2* (Figure 5F), *Myh4* (Figure 5G), or *Myh7* (Figure 5H) in the gastrocnemius.

### 3.6. Effects of HMPA Administration on Myosin Heavy Chain Isoform Expression in Soleus and Gastrocnemius

**Figure 6:**
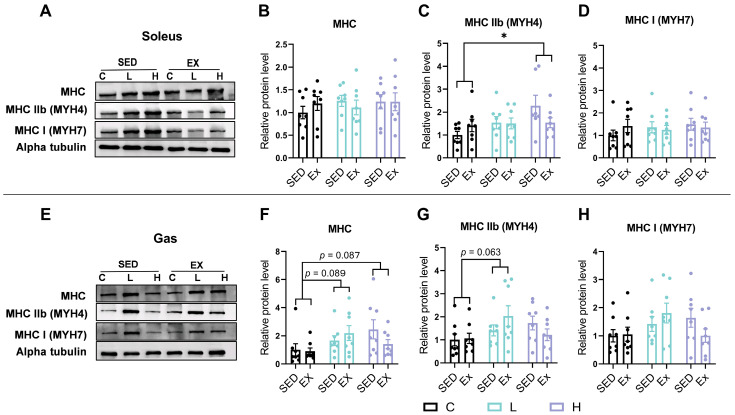
(**A**) Representative Western blot analysis of MHC, MHC IIb, MHC I, and alpha-tubulin in the soleus. Statistical graphs of (**B**) MHC, (**C**) MHC IIb, and (**D**) MHC I protein expression in the soleus. (**E**) Representative Western blot analysis of MHC, MHC IIb, MHC I, and alpha-tubulin in the gastrocnemius. Statistical graphs of (**F**) MHC, (**G**) MHC IIb, and (**H**) MHC I protein expression in the gastrocnemius. MHC, myosin heavy chain; Gas, gastrocnemius; C group, vehicle control group; L group, low-dose HMPA administration group; H group, high-dose HMPA administration group; SED, sedentary; EX, exercise. The values are expressed as the mean ± SEM; * *p* < 0.05 compared with the C group.

Furthermore, we assessed the protein expression of total MHC, MHC IIb, and MHC I in the soleus and gastrocnemius, respectively. Exhaustive exercise did not affect the protein expression levels of MHC, MHC IIb, and MHC I in both the soleus and the gastrocnemius (Figure 6). Consistent with the changes in mRNA expression, the high-dose HMPA administration upregulated the protein expression levels of MHC IIb in the soleus (Figure 6C, *p* < 0.05). However, there were no significant changes in the total MHC (Figure 6B) or MHC I protein expression (Figure 6D). In the gastrocnemius, a trend of increased MHC protein expression was observed with both low-dose HMPA (Figure 6F, *p* = 0.089) and high-dose HMPA administration (Figure 6F, *p* = 0.087). Additionally, an increasing trend in MHC IIb protein expression was observed with low-dose HMPA administration (Figure 6G, *p* = 0.063). No significant changes were observed in MHC I protein expression (Figure 6H).

### 3.7. Effects of HMPA Administration on Insulin-Like Growth Factor 1 Gene Expression in Soleus and Gastrocnemius

**Figure 7:**
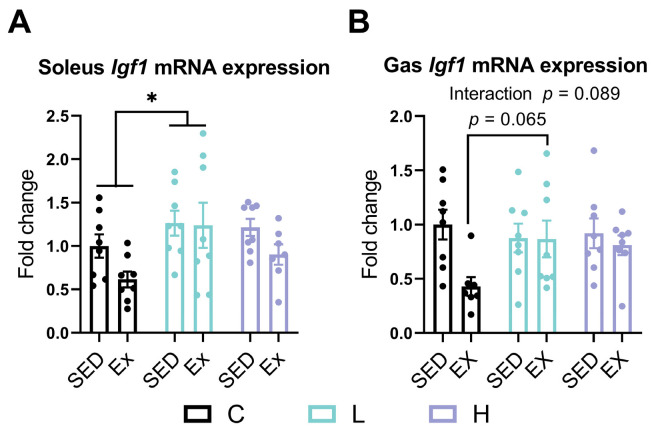
mRNA expression of (**A**) *Igf1* in the soleus and (**B**) *Igf1* in the gastrocnemius. *Igf1*, insulin-like growth factor 1; Gas, gastrocnemius; C group, vehicle control group; L group, low-dose HMPA administration group; H group, high-dose HMPA administration group; SED, sedentary; EX, exercise. The values are expressed as the mean ± SEM; Interaction: interactive effect between HMPA administration and exercise; * *p* < 0.05 compared with the C group.

To explore the underlying mechanisms responsible for muscle improvements induced by the HMPA administration, we examined changes in the expression levels of *Igf1*. In the soleus, the low-dose HMPA administration significantly increased the expression levels of *Igf1* compared to the vehicle control administration (Figure 7A, *p* < 0.05). In the gastrocnemius, exhaustive exercise significantly decreased the expression levels of *Igf1* in the vehicle control group (Figure 7B, *p* < 0.05). However, the low-dose HMPA administration group alleviated this decline compared to the vehicle control group after exhaustive exercise (Figure 7B, *p* = 0.065).

### 3.8. Effects of HMPA Administration on Mitochondrial Biogenesis-Related Gene Expression in Soleus

**Figure 8:**
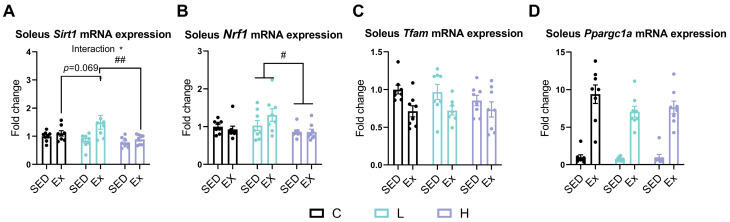
mRNA expression of (**A**) *Sirt1*, (**B**) *Nrf1*, (**C**) *Tfam*, and (**D**) *Ppargc1a* in the soleus. *Sirt1*, sirtuin 1; *Nrf1*, nuclear respiratory factor; *Tfam*, mitochondrial transcription factor a; *Ppargc1a*, peroxisome proliferator-activated receptor gamma coactivator 1-alpha; C group, vehicle control group; L group, low-dose HMPA administration group; H group, high-dose HMPA administration group; SED, sedentary; EX, exercise. The values are expressed as the mean ± SEM; Interaction: interactive effect between HMPA administration and exercise; * *p* < 0.05; # *p* < 0.05 and ## *p* < 0.01 compared with the H group.

Mitochondrial biogenesis plays a key role in enhancing oxidative capacity and influencing the proportion of oxidative muscle fibers [42]. Given the high mitochondrial content of the soleus, we measured the expression of mitochondrial biogenesis-related genes, such as sirtuin 1 (*Sirt1*), nuclear respiratory factor 1 (*Nrf1*), mitochondrial transcription factor A (*Tfam*), and peroxisome proliferator-activated receptor gamma coactivator 1-alpha (*Ppargc1a* or *PGC-1α*), exclusively in this muscle. There was a significant interaction effect between the HMPA administration and exercise on the *Sirt1* expression levels (Figure 8A, *p* < 0.05). The post hoc analysis indicated that the LE group demonstrated significantly elevated *Sirt1* expression compared to the HE group (Figure 8A, *p* < 0.001) and showed a trend toward elevated expression relative to the CE group, though this difference did not reach statistical significance (Figure 8A, *p* = 0.069). Additionally, the expression levels of *Nrf1* were significantly higher in the low-dose HMPA administration group compared to the high-dose HMPA administration group (Figure 8B, *p* < 0.05), indicating that low-dose HMPA may more effectively promote mitochondrial biogenesis than high-dose HMPA. Exhaustive exercise significantly altered the expression levels of *Tfam* (Figure 8C, *p* < 0.001) and *Ppargc1a* (Figure 8D, *p* < 0.0001), whereas the HMPA administration did not cause significant changes.

### 3.9. Effects of HMPA Administration on mTOR/AMPK Phosphorylation in Soleus and Gastrocnemius

**Figure 9:**
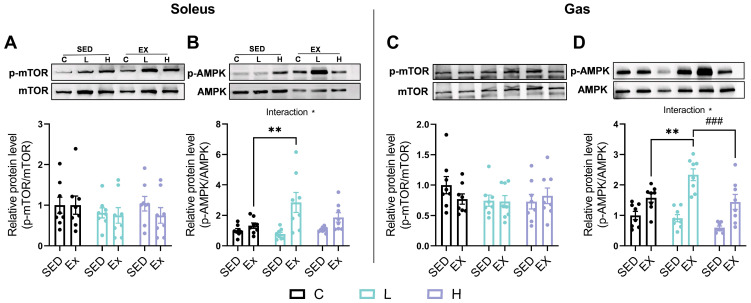
Western blot analysis of the phosphorylation of (**A**) mTOR and (**B**) AMPK in the soleus. Western blot analysis of the phosphorylation of (**C**) mTOR and (**D**) AMPK in the gastrocnemius. AMPK, AMP-activated protein kinase; p-AMPK, phosphorylated AMPK; mTOR, mammalian target of rapamycin; p-mTOR, phosphorylated mTOR; Gas, gastrocnemius; C group, vehicle control group; L group, low-dose HMPA administration group; H group, high-dose HMPA administration group; SED, sedentary; EX, exercise. The values are expressed as the mean ± SEM; Interaction: interactive effect between HMPA administration and exercise; * *p* < 0.05; ** *p* < 0.01 compared with the C group; ### *p* < 0.001 compared with the H group.

No significant differences in mTOR phosphorylation were observed in the soleus or gastrocnemius after the HMPA administration, regardless of exhaustive exercise (Figure 9A,C). However, compared to the vehicle control group, AMPK phosphorylation significantly increased with the low-dose HMPA administration after exercise in the soleus (Figure 9B, *p* < 0.01). In the gastrocnemius, the low-dose HMPA administration resulted in higher AMPK phosphorylation than the high-dose administration after exercise (Figure 9D, *p* < 0.001).

## 4. Discussion

Preliminary evidence reveals that polyphenolic antioxidants may enhance recovery from intensive exercise and subsequently improve physical performance, particularly in cases where substantial exercise-induced muscle damage has occurred [18,43]. In the present study, we employed treadmill exhaustive exercise to evaluate the effects of HMPA on exercise-induced oxidative stress by assessing plasma antioxidant capacity and oxidative stress status through changes in reactive oxygen metabolites, BAP values, OXY-adsorbent levels, and nitrite/nitrate concentration. Our findings show that exhaustive exercise significantly increases the BAP values (Figure 1B) and plasma nitrite/nitrate concentration (Figure 1D) but does not significantly alter the reactive oxygen metabolites (Figure 1A) or OXY-adsorbent levels (Figure 1C), suggesting that exhaustive exercise leads to increased antioxidant defenses and the excessive production of NO. Notably, the oral administration of HMPA at doses of 50 and 500 mg/kg for 14 days significantly attenuated oxidative stress, as evidenced by the lower plasma levels of reactive oxygen metabolites (Figure 1A). Moreover, the high-dose HMPA administration enhanced the total antioxidant capacity and suppressed NO production, as indicated by the increased OXY-adsorbent levels (Figure 1C) and decreased nitrite/nitrate concentration (Figure 1D) following exhaustive exercise. However, the low-dose HMPA administration exhibited a similar, non-significant trend in altering these parameters (Figure 1C,D). These findings suggest that the HMPA administration effectively alleviates oxidative stress and enhances antioxidant capacity post-exercise, potentially in a dose-dependent manner, which may help protect muscle tissue from oxidative damage.

Furthermore, to evaluate the effects of HMPA on oxidative stress in skeletal muscle, we analyzed the MDA content in the soleus (primarily contains slow-twitch fibers) and the gastrocnemius (primarily contains fast-twitch fibers). Previous investigations have revealed differences in the MDA content between these muscles, with higher levels observed in the soleus post-exercise, indicating a greater involvement of the soleus in oxidative metabolism and oxidative stress [44]. Previous studies have demonstrated that HMPA reduces oxidative damage by inhibiting lipid peroxidation, as indicated by decreased 8-isoprostane production [36]. Although numerous studies reported that strenuous exercise can lead to the accumulation of MDA in skeletal muscle [18,45], the present study found no significant changes in the MDA content after exhaustive exercise (Figure 2). Consistent with the present results, Wang et al. also demonstrated that the MDA content of the gastrocnemius remains unchanged immediately after exhaustive exercise but shows a significant increase at 72 h [46]. Furthermore, Sun et al. reported a significant reduction in the MDA content of the gastrocnemius at 0 h, 2 h, 6 h, 12 h, 24 h, and 48 h following acute exhaustive exercise [47]. It is worth noting that the exercise-induced oxidative stress response can vary depending on several factors, including exercise intensity, duration, the sensitivity of the chosen biomarkers, sample tissue, sampling time, and training status [48,49]. These discrepancies in the MDA content following exhaustive exercise may be attributed to differences in exercise protocols, sampling times, and the sensitivity and specificity of MDA as a biomarker for oxidative damage, considering that it represents only one of many byproducts of lipid peroxidation [50]. Thus, the effects of HMPA on exercise-induced oxidative stress require further investigation.

HMPA has been demonstrated to ameliorate oxidative stress by activating the expression of nuclear factor erythroid 2-related factor 2 (Nrf2), which orchestrates the cellular defense mechanism by inducing the transcription of genes encoding antioxidant enzymes [33]. Moreover, HMPA has been shown to restore the TNF-α-induced depletion of glutathione, a critical indicator of intracellular non-enzymatic antioxidant defenses, and significantly reduces the abnormal elevation of GPx and glutathione reductase activities in a dose-dependent manner [51]. In the present study, we analyzed the antioxidant enzyme expression in both soleus and gastrocnemius because of their distinct oxidative properties. The soleus, a slow-twitch muscle, displays higher antioxidant enzyme activities (e.g., CAT and SOD), providing greater protection against oxidative stress compared to fast-twitch muscles like the gastrocnemius [44,52]. In contrast, the gastrocnemius produces more H_2_O_2_ at rest and generates higher levels of ROS during exercise, making it more susceptible to oxidative damage [44,52]. Antioxidant enzymes such as SOD1, SOD2, and CAT are considered the primary line of defense in the antioxidant system to neutralize ROS. SOD1 and SOD2 convert O_2_^−^ into H_2_O_2_ in the cytoplasm and mitochondria, respectively, while CAT further breaks down H_2_O_2_ into water and oxygen [53,54,55]. NQO1 is considered the phase II detoxifying enzyme that reduces quinones and enhances cellular defense [56]. Our study found that high-dose HMPA selectively modulated antioxidant enzyme expression in the soleus by increasing *Sod1* expression (Figure 3A) while suppressing *Nqo1* expression (Figure 3D). Unexpectedly, low-dose HMPA did not significantly affect antioxidant enzyme expression (Figure 3) despite reducing the plasma reactive oxygen metabolite levels (Figure 1A). This suggests that the oxidative stress reduction observed with low-dose HMPA may rely more on direct antioxidant activity than on upregulating endogenous antioxidant enzymes.

To explore the mechanism underlying the reduction in the plasma NO levels after the HMPA administration, we examined the mRNA expression levels of NOS in muscle tissues (Figure 4). Substantial evidence indicates that increased iNOS expression leads to excessive NO production, contributing to oxidative stress and cellular damage [57]. In contrast, eNOS generally enhances vascular function and NO bioavailability, alleviating exercise-induced oxidative stress and promoting muscle recovery [58]. Previous research demonstrated that HMPA can inhibit iNOS expression and the resulting excessive NO production caused by lipopolysaccharide treatment [33]. Our results showed that the high-dose HMPA administration suppressed the mRNA abundance of *Nos2* (*iNOS*) (Figure 4A) and *Nos3* (*eNOS*) (Figure 4B) in the soleus. Similarly, the high-dose HMPA administration reduced *Nos2* (*iNOS*) mRNA abundance in the gastrocnemius (Figure 4C), which may help explain the observed reduction in the plasma NO levels. However, the suppression of NOS expression in both the soleus and gastrocnemius muscles raises potential concerns, as NOS enzymes play a crucial role in activating acute and adaptational mechanisms, as well as facilitating muscle fiber phenotypes transitions through the generation of NO [59,60]. Numerous studies have documented that inhibiting NOS activity impedes the conversion of muscle fibers from fast-twitch to slow-twitch types and promotes the expression of fast-twitch fibers [60,61]. Although this shift may enhance power and speed, it is generally unfavorable for endurance performance due to the reduction in fatigue resistance typically associated with slow-twitch fibers. These findings highlight the complexity of HMPA administration on redox signaling: on the one hand, high-dose HMPA can mitigate oxidative stress; on the other hand, it may interfere with the recovery pathways reliant on NO signaling.

Polyphenolic antioxidants can preserve muscle fiber type and facilitate fiber type transitions by modulating redox signaling pathways. Epigallocatechin gallate treatment has been found to inhibit the formation of slow-twitch fibers by reducing ROS levels without impacting the expression of fast-twitch fibers [62]. CGA can be supplemented to improve meat quality and modulate muscle fiber composition. Supplementation with 0.04% CGA for 35 days has been observed to promote a shift towards more oxidative muscle fibers (Type I and Type IIa) in the longissimus dorsi muscle, as evidenced by the upregulation of myogenic factors such as myogenic differentiation 1 and myogenin [23]. Furthermore, CGA supplementation increased the expression of antioxidant genes, including *Nrf2* and *Gpx1*, suggesting enhanced antioxidant capacity within muscle tissue, which contributes to the observed improvements in muscle quality and resilience [23]. As a gut metabolite of CGA, HMPA may retain some effects similar to those of CGA. In our previous study, a 14-day HMPA administration suggested a potential role in promoting myogenesis by upregulating the expression of myogenic regulatory factor 5 [38]. However, its effects on muscle fiber type expression remained unclear. The current findings reveal that the mRNA expression of *Myh4*, which encodes the MHC IIb isoform, was significantly increased in the soleus after 14 days of low-dose HMPA administration, with a similar trend observed at high doses (Figure 5C). Additionally, high-dose HMPA led to an increase in MHC IIb protein expression in the soleus (Figure 6C). In the gastrocnemius, both the total MHC and MHC IIb protein expression showed an increasing trend following low-dose HMPA administration, whereas high-dose HMPA showed a similar trend only in the total MHC expression (Figure 6F,G). However, these changes were not detected at the mRNA level in the gastrocnemius (Figure 5E–H). The discrepancy between the mRNA and protein expression levels may result from post-transcriptional regulation, differences in protein turnover rates, or variations in translational efficiency [63]. In summary, HMPA administration promotes the formation of Type IIb fast fibers and exerts a more pronounced effect on the soleus muscle compared to the gastrocnemius.

Muscle performance can be influenced by the distribution of muscle fiber types. The enhanced force-generating capacity is attributable to the greater abundance of fast-twitch fibers, whereas the superior endurance performance and fatigue resistance are associated with a proportional increase in slow-twitch fibers [64,65]. In our previous study, HMPA administration has been shown to enhance grip strength in mice, possibly due to fast-twitch fiber hypertrophy [38]. Type IIb fibers, the most glycolytic subtype of fast-twitch fibers, are capable of generating exceptionally rapid and powerful contractions [7]. In the present study, we further demonstrated that HMPA administration enhances the expression of both MHC IIb mRNA (Figure 5C) and protein levels (Figure 6C), further elaborating on the perspective proposed earlier. These findings shed light on the potential applications of HMPA for individuals with poor muscle strength and selective loss of fast-twitch fibers. For example, aging is known to cause a selective decline in fast-twitch fibers while sparing slow-twitch fibers. HMPA shows potential as a nutritional supplement for combating age-related selective muscle atrophy, particularly by enhancing the hypertrophic growth of fast-twitch fibers.

To further explore the molecular mechanisms by which HMPA influences muscle growth and adaptations, we analyzed the expression of growth factors such as IGF-1, transcription factors involved in mitochondrial biogenesis, and the activity of mTOR and AMPK. It is widely accepted that IGF-1 is a well-characterized growth factor, acting upstream to activate several anabolic signaling pathways. IGF-1 can counteract the negative regulator myostatin, thereby stimulating muscle mass growth by promoting myoblast proliferation [66]. Our findings suggest that HMPA plays a significant role in regulating *Igf1* expression in skeletal muscles (Figure 7). Specifically, the low-dose HMPA administration significantly upregulated *Igf1* mRNA expression in the soleus (Figure 7A), suggesting a potential anabolic response that may promote the hypertrophy of fast-twitch fibers through the IGF-1 pathway. Recent reports have shown that epigallocatechin-3-gallate treatment enhances gastrocnemius muscle mass in aged rats by upregulating the expression of *Il15* and *Igf1* while inhibiting the expression of myostatin, as well as E3 ubiquitin ligases, such as MuRF1 and MAFbx [67]. The Gu-Shu-Kang capsule has been shown to enhance muscle mass and function in dexamethasone-treated mice, potentially by mitigating the upregulation of atrogin-1(MAFbx) and MuRF-1 through the activation of the IGF-1 signaling pathway [68]. In the present study, the low-dose HMPA administration prevented the decline in *Igf1* expression caused by exhaustive exercise in the gastrocnemius muscle (Figure 7B). Additionally, our previous research demonstrated that HMPA administration suppresses protein catabolism induced by exhaustive exercise in this muscle, as indicated by the inhibition of *Trim63* (*MuRF1*) and *Map1lc3b* (*LC3b*) expression after exercise [38]. This suppressive effect of HMPA on these catabolic pathways may also be attributed to the upregulation of *Igf1*.

Mitochondria support the metabolic activity, growth, and regeneration of skeletal muscles, with mitochondrial dysfunction being associated with a reduction in oxidative capacity and a decline in fatigue resistance [69,70]. Thus, mitochondrial biogenesis is essential for promoting changes in substrate utilization, muscle fiber composition, and exercise performance. SIRT1, a NAD^+^-dependent deacetylase, regulates mitochondrial biogenesis and energy metabolism through the deacetylation of PGC-1α [71]. In turn, PGC-1α co-activates Nrf1, thereby enhancing the transcription of mitochondrial genes, including TFAM, which is essential for mitochondrial DNA replication and transcription [72]. SIRT1 and PGC-1α play an important role in regulating muscle fiber composition [23]. As demonstrated by Chen et al., HMCA shifts muscle composition toward oxidative, slow-twitch fibers (Type I and Type IIa) by activating PGC-1α expression through the SIRT1/AMPK signal pathway [21]. In addition, it is a widely held view that SIRT1 and PGC-1α are highly upregulated in skeletal muscles after physical exercise in order to enhance mitochondrial biogenesis and meet energy demands [73,74]. Consistent with this view, our study shows that exhaustive exercise significantly increased the expression of *Sirt1* (Figure 8A) and *Ppargc1a* (*PGC-1α*) (Figure 8D). The low-dose HMPA administration enhanced post-exercise *Sirt1* expression compared to both the vehicle control and high-dose HMPA groups (Figure 8A). However, this alteration was not observed in the expression of *Ppargc1a* (*PGC-1α*) (Figure 8D). Moreover, the low-dose HMPA administration significantly increased *Nrf1* expression relative to the high-dose HMPA group (Figure 8B). These results suggest that HMPA administration, particularly at low doses, may promote muscle physiological adaptation by enhancing mitochondrial biogenesis.

AMPK is a key metabolic sensor that maintains muscle performance and supports adaptive responses to exercise by promoting mitochondrial biogenesis and optimizing energy metabolism [75]. Additionally, AMPK and SIRT1 exert mutually reinforcing effects and collaborate to enhance mitochondrial quality, maintain cellular homeostasis, and promote cell survival [76]. Our results show that the low-dose HMPA administration significantly increased the phosphorylation of AMPK after exercise compared to the vehicle control in both the soleus (Figure 9B) and gastrocnemius (Figure 9D). These findings suggest that low-dose HMPA may improve energy utilization and facilitate muscle adaptations during prolonged exercise through the activation of the AMPK/SIRT1 pathway. However, the high-dose HMPA administration failed to exhibit comparable regulatory effects (Figure 8 and Figure 9), possibly due to its strong antioxidant activity in scavenging ROS, a key signaling factor in muscle adaptation. This observation aligns with recent evidence suggesting that high doses of antioxidants may blunt molecular adaptations and diminish exercise benefits by excessively eliminating ROS [77,78]. mTOR, counteracting AMPK activity, is responsible for promoting anabolic processes and supporting cell growth [79]. A 30-day supplementation with HMCA in adult zebrafish has been reported to activate the mTOR pathway, contributing to the hypertrophic growth of fast-twitch muscle and muscle mass [35]. Unexpectedly, HMPA did not affect mTOR expression (Figure 9A,C), indicating that its influence on muscle development may instead be mediated through the regulation of *Igf1* expression.

Despite the promising results, this study had several limitations. First, the sample size was limited. Second, the administration period in this experiment was relatively short. To improve clinical applicability, future studies should investigate the long-term effects and optimal dosage of HMPA to evaluate its safety and potential adverse effects over extended use. Third, due to the limited quantity of available muscle samples, this study did not include an analysis of the morphological changes in muscle fiber composition and mitochondrial content, warranting further investigation into the effects of HMPA on muscle mass and muscle fiber morphology. Future studies should make efforts to address these limitations and explore the broader applications of HMPA in animal models or clinical trials, focusing on its potential role in mitigating age-related muscle loss and other chronic conditions associated with oxidative stress, such as metabolic disorders and cardiovascular diseases.

## 5. Conclusions

In summary, our findings demonstrate that both low and high doses of 14-day HMPA administration reduce baseline oxidative stress, whereas only high-dose HMPA further decreases plasma nitrite/nitrate concentration and enhances post-exercise antioxidant capacity. This effect appears to be dose-dependent, potentially mediated by the modulation of antioxidant enzyme activity and the suppression of NOS expression in skeletal muscle, which may further influence muscle fiber phenotype expression. In addition, our results suggest that HMPA promotes the formation of fast-twitch fibers and muscle adaptation through the activation of IGF-1 and the AMPK/SIRT1 pathway. Notably, these functional effects of HMPA vary depending on the dose, with high-dose HMPA exhibiting stronger antioxidant properties, while low-dose HMPA is more effective in promoting muscle growth and facilitating post-exercise muscle adaptation. These dose-dependent effects warrant further investigation into the efficacy, optimal dosage, and duration of HMPA supplementation in different contexts. For instance, low-dose HMPA may aid in enhancing post-exercise recovery in athletes or preserving muscle mass in aging populations, whereas high-dose HMPA may be more effective in reducing oxidative stress and inflammation in chronic conditions, such as diabetes-related inflammatory myopathy.

## Figures and Tables

**Table 1:** Primer sequences for qPCR analysis.

Gene GenBank Accession No.	Forward	Reverse
*Myh1* NM_030679	AGAAGCTCCTGGGATCCATT	CTCTCGCCAAGTACCCTCTG
*Myh2* NM_00103954	GAGCAAAGATGCAGGGAAAG	TAAGGGTTGACGGTGACACA
*Myh4* NM_010855	GGGGCTGTACCAGAAATCCG	CCTGAAGAGAGCTGACACGG
*Myh7* NM_080728	AGATGAATGCCGAGCTCACT	CTCATCCAAACCAGCCATCT
*Sod1* NM_011434	GAACCAGTTGTGTTGTCAG	GTACAGCCTTGTGTATTGTC
*Sod2* NM_013671	CAACTCAGGTCGCTCTTC	TGATAGCCTCCAGCAACT
*Cat* NM_009804	GATGGAGAGGCAGTCTATTG	ATTGGCGATGGCATTGAA
*Nqo1* NM_008706	CGAATCTGACCTCTATGCTAT	GCGTCCTTCCTTATATGCTA
*Nos2* NM_010927	GCAAACCCAAGGTCTACGTTCA	GAGCACGCTGAGTACCTCATTG
*Nos3* NM_008713	GGCTCTCACCTACTTCCT	GGCTCTCACCTACTTCCT
*Igf1* NM_010512	CGCTCTGCTTGCTCACCTTCAC	CCTCGGTCCACACACGAACTGA
*Sirt1* NM_019812	ACGGTATCTATGCTCGCCTTGC	GACACAGAGACGGCTGGAACTG
*Nrf1* NM_010938	GTGGGACAGCAAGCGATTGTAC	GTGGGACAGCAAGCGATTGTAC
*Tfam* NM_009360	GTGGGACAGCAAGCGATTGTAC	CTTCAGCCATCTGCTCTTCC
*Ppargc1a(Pgc-1alpha)* NM_008904	ACACAACCGCAGTCGCAACAT	GCAGTTCCAGAGAGTTCCACACTT
*Rps18* NM_011296	TTCTGGCCAACGGTCTAGACAAC	CCAGTGGTCTTGGTGTGCTGA

*Myh1*, myosin, heavy polypeptide 1, skeletal muscle, adult; *Myh2*, myosin, heavy polypeptide 2, skeletal muscle, adult; *Myh4*, myosin, heavy polypeptide 4, skeletal muscle; *Myh7*, myosin, heavy polypeptide 7, cardiac muscle, beta; *Sod*, superoxide dismutase; *Cat*, catalase; *Nqo1*, NAD(P)H dehydrogenase, quinone 1; *Nos2* (*iNOS*), nitric oxide synthase 2, inducible; *Nos3* (*eNOS*), nitric oxide synthase 3; *Igf1*, insulin-like growth factor 1; *Sirt1*, sirtuin 1; *Nrf1*, nuclear respiratory factor 1; *Tfam*, mitochondrial transcription factor a; *Ppargc1a*, peroxisome proliferator-activated receptor gamma coactivator 1-alpha; *Rps18*, ribosomal protein S18.

## Data Availability

Data are contained within the article.

## Abbreviations

The following abbreviations are used in this manuscript:

MHC	myosin heavy chain
ROS	reactive oxygen species
RNS	reactive nitrogen species
NO	nitric oxide
SOD	superoxide dismutase
CAT	catalase
GPx	glutathione peroxidase
HMPA	3-(4-Hydroxy-3-methoxyphenyl)propionic acid
HMCA	4-Hydroxy-3-methoxycinnamic acid
CGA	chlorogenic acid
d-ROMs	diacron-reactive oxygen metabolites
BAP	biological antioxidant potential
NOS	nitric oxide synthase
NQO1	NAD(P)H dehydrogenase
IGF-1	insulin-like growth factor 1
SIRT1	sirtuin 1
NRF1	nuclear respiratory factor 1
TFAM	mitochondrial transcription factor a
PGC-1α	peroxisome proliferator-activated receptor gamma coactivator 1-alpha
AMPK	AMP-activated protein kinase α
mTOR	mechanistic target of rapamycin
NRF2	nuclear factor erythroid 2-related factor 2
